# Expression and significance of histone H3K27 demethylases in renal cell carcinoma

**DOI:** 10.1186/1471-2407-12-470

**Published:** 2012-10-12

**Authors:** Yongqing Shen, Xiaoqiang Guo, Yuejia Wang, Wei Qiu, Yanzhong Chang, Aili Zhang, Xianglin Duan

**Affiliations:** 1 Laboratory of Iron Metabolism and Molecular Biology, College of Life Science, Hebei Normal University, Shijiazhuang, 050016, China; 2 Department of Biochemistry, Bethune Military Medical College, Shijiazhuang, 050081, China; 3 Department of Urology, The Fourth Hospital, Hebei Medical University, Shijiazhuang, 050011, China; 4 Department of Urology, Peking University First Hospital, Beijing, 100034, China; 5 Xishan College, Hebei Medical University, Shijiazhuang, 050011, China

**Keywords:** Renal cell carcinoma, Histone H3K27 demethylase, UTX, JMJD3, Epigenetics

## Abstract

**Background:**

The histone H3K27 demethylases UTX and JMJD3 are important regulatory factors that modulate gene expression by altering the physical state of chromatin. Previous studies have indicated an abnormal H3K27 methylation status in carcinogenesis. We therefore investigated the expression patterns of UTX and JMJD3 in renal cell carcinoma (RCC) and their roles in cancer development.

**Methods:**

The mRNA expression levels of the *UTX* and *JMJD3* genes were determined in cancer tissues and adjacent normal tissues in 36 patients with primary RCC, using quantitative real-time-polymerase chain reaction. The UTX and JMJD3 protein contents were measured by western blotting and immunohistochemical analysis.

**Results:**

*UTX* and *JMJD3* transcripts were significantly increased in cancer tissues compared to normal tissues (P < 0.05). mRNA levels of the inhibitor of cyclin-dependent kinases 4 and 6 *p16INK4a* were also increased in cancer tissues (P < 0.001). Western blotting indicated that levels of both demethylases were increased in cancer tissues. The level of tri-methylated H3K27 (H3K27me3) was lower in cancer tissues compared to normal tissues, but expression of the H3K27 methyltransferase EZH2 was increased (P < 0.05). These results suggest that the two H3K27 demethylases may play critical roles in the regulation of H3K27 methylation status in RCC. Immunohistochemical analysis confirmed that UTX and JMJD3 expression were upregulated in cancer tissues compared to adjacent tissues.

**Conclusions:**

This study demonstrated that UTX and JMJD3 were upregulated in cancer tissues, suggesting that they may be involved in the development of primary RCC. The potential roles of H3K27 demethylases as biomarkers in the early diagnosis of RCC need to be further explored.

## Background

Kidney cancer is the third most common urological malignancy and is responsible for an estimated 120,000 deaths per year worldwide [1]. The incidence of kidney cancer has increased significantly over the past 20 years [2], and accounts for nearly 3% of all cancer cases in Europe [3]. The two most common types of kidney cancer are renal cell carcinoma (RCC) and urothelial cell carcinoma. RCC is the most common kidney cancer in adults, and is generally resistant to chemotherapy and radiation therapy [4,5]. Radical or partial nephrectomy of the tumor at a localized stage remains the mainstay of curative therapy [6]. Unfortunately, however, distant metastases are present at the time of initial diagnosis in approximately one third of patients, and the tumor will recur in a further third, even after nephrectomy with curative intent [7,8]. In addition, there is lack of specific diagnostic markers for RCC, which is an important contributory factor in its poor prognosis [9]. A better understanding of the molecular basis of RCC has facilitated the development of novel and more selective diagnostic and therapeutic approaches.

Many studies have shown that epigenetic modification plays an important role in cancer development [10]. Histone methylation is essentially a post-translational and epigenetic modification, which is miswritten, misinterpreted and incorrectly erased in many human cancers, including RCC [11]. Histone methylation can occur at certain amino acids including lysine (K) and arginine (R) in the N-terminus of histones H3 and H4, by the addition of one, two, or three methyl groups. H3K27 methylation is a reversible process that can be catalyzed by histone lysine methyltransferase 6 (KMT6, also known as EZH2), and the demethylases 6A (KDM6A, also known as ubiquitously-transcribed *TPR* gene on the X chromosome, UTX) and 6B (KDM6B, also known as jumonji domain-containing protein 3, JMJD3) [12]. Tri-methylated histone H3K27 (H3K27me3) is a suppressor marker that inhibits the expression of specific target genes by altering the physical state of chromatin. *EZH2* (enhancer of zeste homolog 2) can be regarded as an oncogene and is frequently overexpressed in a wide variety of cancers [13], including RCC [14,15]. Several studies have identified UTX and JMJD3 as H3K27-specific demethylases that contribute to gene activation [16-20]. Both UTX and JMJD3 are considered as tumor suppressors, and inactivating somatic mutations of UTX are frequently observed in several tumor types, including RCC [21]. However, the relationship between the expression of these two enzymes and cancer development is largely unknown. In this study, we therefore investigated the gene and protein expression levels of UTX and JMJD3 and their clinical significance in RCC.

## Methods

### Patients and tissue specimens

Sixty-three patients were diagnosed with clear cell RCC by computed tomography (CT) combined with clinical symptoms, and the diagnoses were confirmed by clinicopathological examination at the Fourth Clinical Medical College of Hebei Medical University (Shijiazhuang, China). All cancer tissues and paired adjacent tissues were obtained through radical nephrectomy. Written informed consent was obtained from all patients before surgery and the study was approved by the Human Research Ethics Committee of The Fourth Clinical Medical College of Hebei Medical University. Histological classification (Additional file 1: Figure S1) was performed according to the standard provided by Fuhrman et al. [22] and postoperative pathological staging was performed in all cases (Table 1). 

**Table 1:** Demographics of patients and tumor characteristics of radical nephrectomy specimens

**Characteristics**		**Number**
Patients Age (year)	Mean	60.3
	Range	33~79
Gender	Male	48
	Female	15
Tumor stage	T1a	21
	T1b	19
	T2	16
	T3/T4	7
Tumor size (cm)	Mean	4.2
	Range	1.1~10.5
Tumor location	Side (left/right)	29L/34R
	Upper pole	33
	Middle pole	19
	Lower pole	10
Fuhrman grading	Grade 1	31
	Grade 2	25
	Grade 3	4
	Grade 4	3
Lymph node	Negative	63
	Positive	0

### Quantitative real-time-polymerase chain reaction (qRT-PCR)

Total RNA was extracted from cancer tissues and adjacent tissues with Trizol reagent (Invitrogen, Carlsbad, CA, USA) according to the manufacturer’s protocol. The total RNA concentration was determined using a NanoDrop ND-1000 spectrophotometer (Thermo Scientific, Wilmington, DE, USA). cDNA was synthesized from 2 μg of total RNA using a RT system, according to the manufacturer’s instructions (Invitrogen). The mRNA expression levels of *UTX*, *JMJD3*, *EZH2* and *p16INK4a* were analyzed using SYBR green PCR Mix (Tiangen, Beijing, China), with 18S rRNA as an internal reference. qRT-PCR was performed using a 7500 RealTime PCR System (Applied Biosystems, Foster City, CA, USA). Primer sequences were synthesized by Sangon (Shanghai, China) and included: *UTX* forward 5’- TTTGTCAATTAGGTCACTTCAACCTC −3’ and *UTX* reverse 5’- AAAAAGGCAGCATTCTTCCAGTAGTC −3’, *JMJD3* forward 5’- GGAGGCCACACGCTGCTAC −3’ and *JMJD3* reverse 5’- GCCAGTATGAAAGTTCCAGAGCTG-3’, *EZH2* (isoforms 1–5) forward 5’- GGGACAGTAAAAATGTGTCCTGC-3’ and *EZH2* reverse 5’-TGCCAGCAATAGATGCTTTTTG-3’, *INK4A* (isoforms 1/2) forward 5’- GAAGGTCCCTCAGACATCCCC −3’ and *INK4A* reverse 5’- CCCTGTAGGACCTTCGGTGAC −3’, *18S* rRNA forward 5’-CGGCGGCTTT GGTGACTCTAG-3’ and *18S* rRNA reverse 5’-CCGTTTCTCAGGCTCCCTCTCC-3’. Relative expression levels of the four genes were normalized to the internal reference 18S RNA. Data were analyzed using the comparative threshold cycle (2^-ΔCT^) method.

### Western blotting

Cancer tissues and adjacent normal tissues from all 63 patients were homogenized in radioimmunoprecipitation assay buffer containing the protease inhibitors phenylmethylsulfonyl fluoride (100 μg/mL), NaVO_3_ (1 mmol/L) and dithiothreitol (0.5 mmol/L). Homogenates were centrifuged and supernatants were collected. Protein concentrations were determined using a NanoDrop ND-1000 and corrected appropriately. A total of 50 μg of protein from each sample was resolved by reducing loading buffer and separated by 8% sodium dodecyl sulfate-polyacrylamide gel electrophoresis followed by electrophoretic transfer to a nitrocellulose (NC) membrane. The NC membrane was saturated with 5% skim milk in TBST (50 mM Tris–HCl, 150 mM NaCl, 0.1% Tween-20) for 2 h and then incubated with primary antibodies at 4°C overnight. The primary antibodies used included rabbit polyclonal antibodies to UTX (1:1,000, Abcam, Hong Kong, China), JMJD3 (1:1500, Abcam), EZH2 (1:500, Santa Cruz Biotechnology, Hong Kong, China), H3K27me3 (1:1,500, Epigentek, Brooklyn, USA), H3 (1:2,000, Sigma-Aldrich, St Louis, USA) and actin (1:2,500, Sigma, St Louis, USA). NC membranes were incubated with 1:5,000-diluted peroxidase-coupled goat anti-rabbit immunoglobulin G (IgG) (secondary antibody, Sigma) for 1 h, after washing three times with TBST (5 min/time) at room temperature. After further washing with TBST four times, the NC membranes were exposed to enhanced chemiluminescence substrate (Pierce, Rockford, USA) for 5 min and detection was performed using a Fujifilm LAS-4000 imaging system (GE Healthcare, Bucks, UK).

### Immunohistochemical analysis

After fixation in 4% formalin, cancer tissues and adjacent normal tissues from the 63 RCC patients were dehydrated through an ascending series of graded ethanols, embedded in paraffin wax, and cut into 5-μm sections using a microtome. The endogenous peroxidase activity of sections was inhibited by treatment with 3% H_2_O_2_/methanol. Antigen retrieval was performed on xylene-deparaffinized and dehydrated sections by heating the slides for 10 min in 0.01 M citrate buffer (pH 6.0). Non-specific binding was blocked by incubating sections with 5% BSA in a humidified chamber. Sections were then incubated overnight at 4°C with 1:100 dilution of anti-UTX or anti-JMJD3 primary polyclonal rabbit antibodies (Abcam). After washing twice in PBS, sections were treated with peroxidase-conjugated AffiniPure goat anti-rabbit IgG (ZSGB Bio, Beijing, China) at room temperature for 30 min, followed by diaminobenzidine (ZSGB Bio) as a chromogen to visualize the peroxidase activity. A negative immunohistochemical control was provided by replacement of the primary antibodies by antibody diluents.

The protein expression scores for both UTX and JMJD3 were quantitated according to Wu et al. [23]. Briefly, the proportions of UTX/JMJD3-expressing tumor cells were scored as follows: 0, no positive cells; 1, <5%; 2, 6–25%; 3, 26–50%; 4, 51–75%; and 5, >75%. Staining intensity was graded according to the mean optical density: 0, no staining; 1, weak staining (light yellow); 2, moderate staining (yellow-brown); and 3, strong staining (brown). The staining index was calculated as the product of the staining intensity score and the proportion of UTX/JMJD3-positive tumor cells.

### Statistical analysis

Statistical analysis was carried out using the SPSS 17.0 statistical software package. qRT-PCR and immunohistochemical data were analyzed by two-tailed paired-sample *t*-tests and Mann–Whitney *U* tests (α = 0.05). A P value of ≤ 0.05 was considered to indicate a statistically significant difference between cancer tissues and adjacent normal tissues.

## Results

### Patient clinical characteristics

A total of 63 samples of cancer tissues and paired adjacent normal tissues were available from patients with RCC who had undergone surgery. All the patients were treated by radical nephrectomy and received no preoperative radiation or chemotherapy y. Most patients (56/63) were at an early stage (stages 1 and 2), and no lymph node metastasis was present in any patients. The overall 5-year survival rate was 100%, suggesting that early diagnosis and surgical removal of the cancer tissue resulted in a good prognosis. The clinical data are shown in Table 1.

### mRNA expression levels of UTX and JMJD3 in cancer tissues and adjacent normal tissues in RCC patients

**Figure 1:**
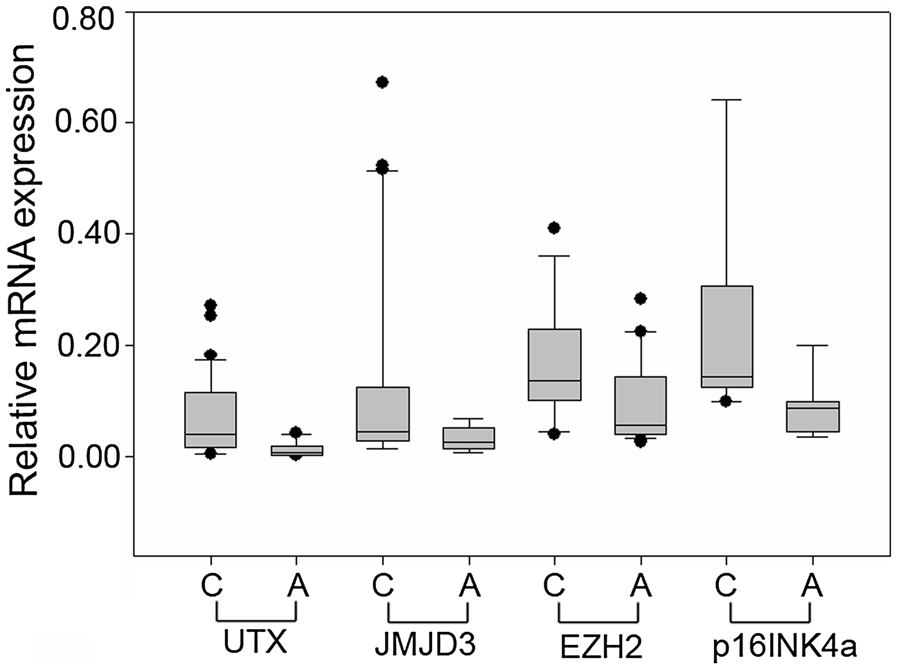
**Real-time qRT-PCR analysis of the H3K27 demethylases *UTX* and *JMJD3*, the H3K27 methyltransferase *EZH2* and the CDK4/CDK6 inhibitor p16INK4a.** Relative mRNA expression levels of *UTX*, *JMJD3* and *EZH2* were higher in RCC cancer tissues than in paired adjacent normal tissues (n = 36; all P < 0.05). The mRNA level of *p16INK4a* was also upregulated in cancer tissues compared to adjacent normal tissues (P < 0.001).

The transcription levels of the two H3K27 demethylase genes, *UTX* and *JMJD3*, the H3K27 methyltransferase *EZH2* and the CDK4/CDK6 inhibitor *p16INK4a* were determined by qRT-PCR in cancer tissues and adjacent normal tissues from 36 RCC patients. The mRNA levels of *UTX*, *JMJD3* and *EZH2* were significantly increased in cancer tissues compared to the adjacent tissues (P < 0.05, Figure 1), while the expression of *p16INK4a* mRNA was highly significantly upregulated in cancer tissues (P < 0.001, Figure 1).

### Levels of UTX and JMJD3 proteins in cancer tissues and adjacent normal tissues in RCC patients

**Figure 2:**
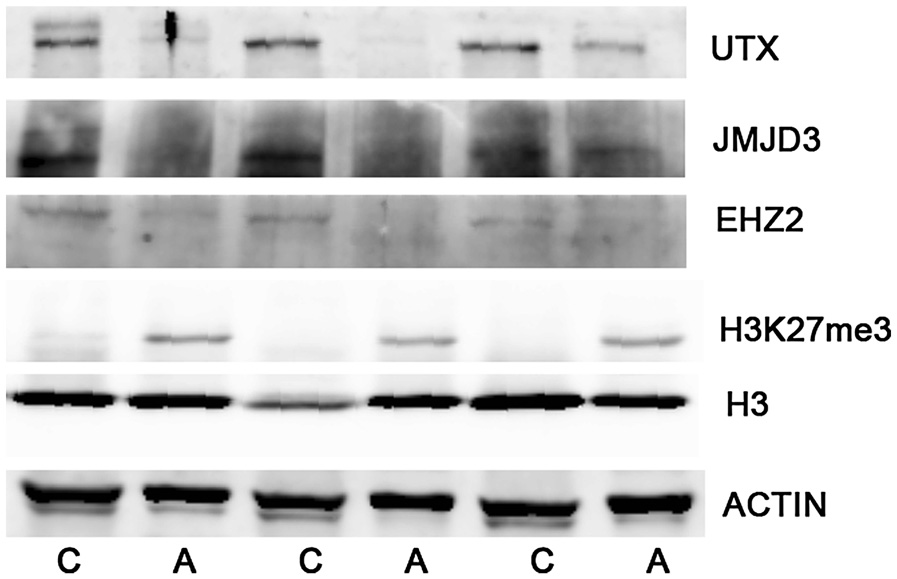
**Western blotting analysis of H3K27 methylation-modifying enzymes and H3K27me3 levels.** The protein expression levels of the H3K27 demethylases UTX and JMJD3 were higher in RCC cancer tissues (C) than in paired adjacent normal tissues (A). The H3K27me3 level was lower in RCC cancer tissues, although the protein level of the H3K27 methyltransferase EZH2 was increased.

To support the changes in mRNA levels of UTX and JMJD3, the protein contents of the two H3K27 demethylases were measured by western blotting. Levels of UTX and JMJD3 proteins were obviously increased in cancer tissues compared to adjacent normal tissues (Figure 2), while the level of H3K27me3 was reduced and H3K27 methyltransferase EZH2 was upregulated (Figure 2).

### Changes in UTX and JMJD3 expression in cancer tissues and adjacent normal tissues in RCC patients analyzed by immunohistochemistry

**Figure 3:**
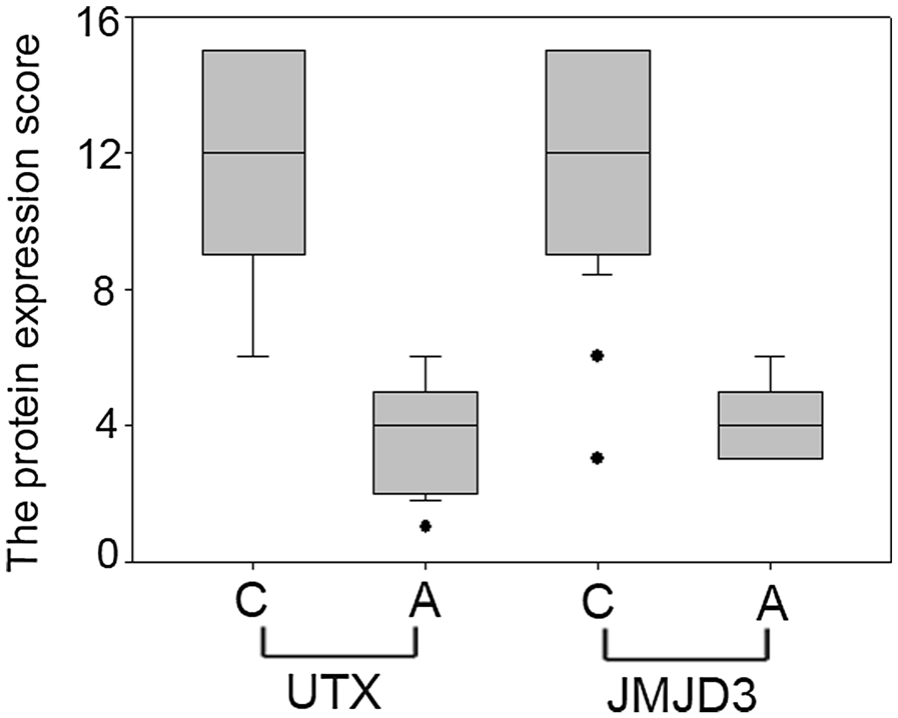
**Changes in UTX and JMJD3 protein expression levels in RCC.** Levels of UTX and JMJD3 proteins were higher in RCC cancer tissue samples (C) than in paired adjacent normal tissue samples (A) (n = 63, P < 0.05).

**Figure 4:**
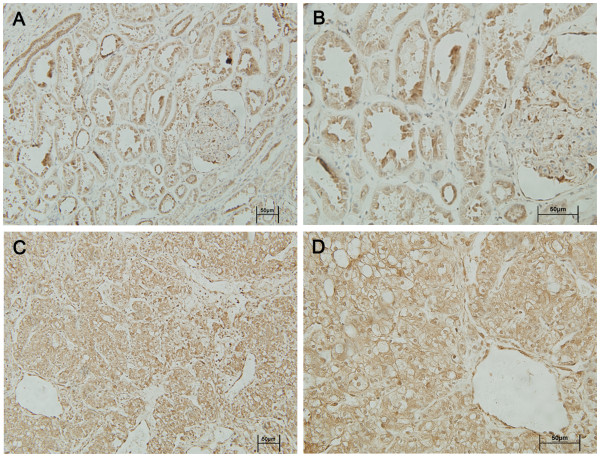
**Immunohistochemical analysis of UTX expression.** UTX protein expression was obviously higher in cancer tissues (**C** and **D**) than in adjacent normal tissues (A and B). Magnifications × 200 (**A** and **C**) and × 400 (B and D).

**Figure 5:**
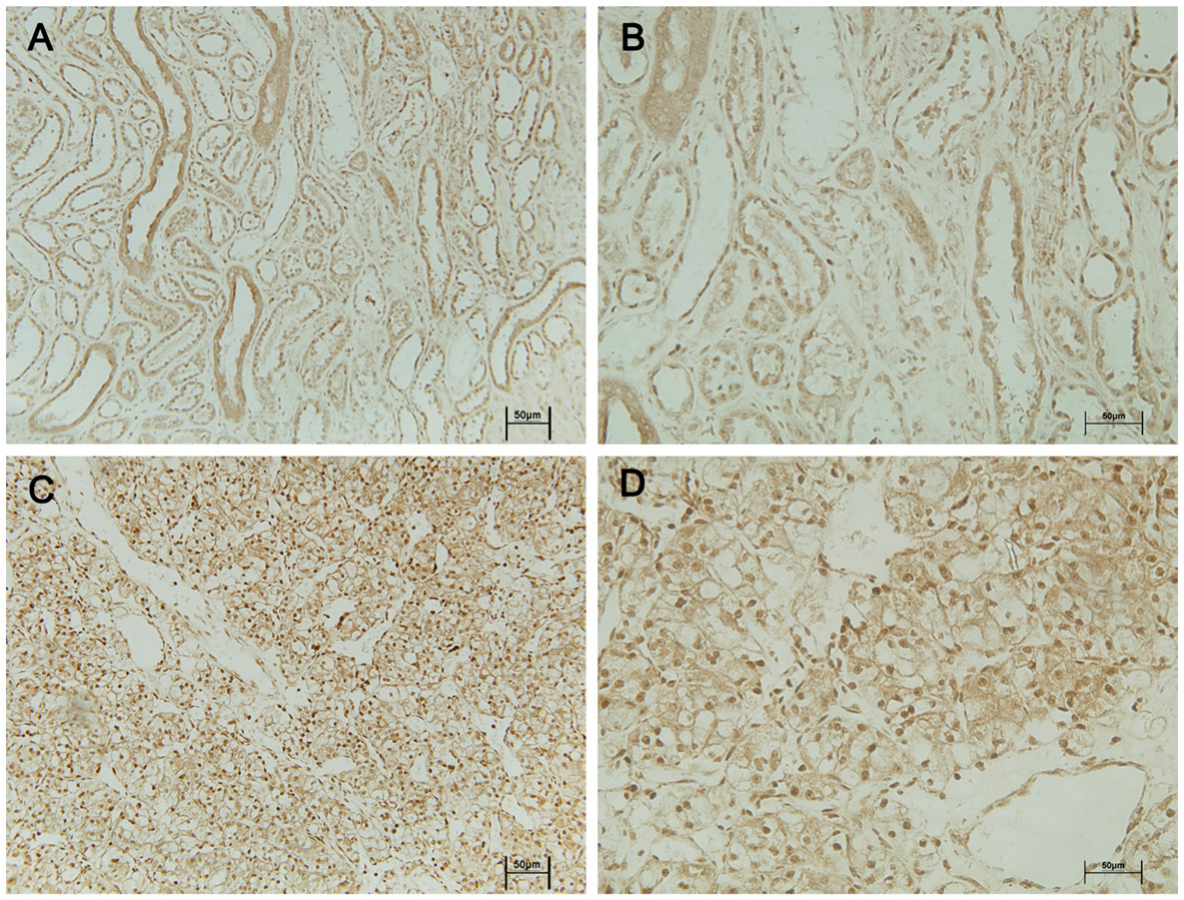
**Immunohistochemical analysis of JMJD3 expression.** JMJD3 protein expression was obviously higher in cancer tissues (**C** and **D**) than in adjacent normal tissues (**A** and **B**). Magnifications × 200 (**A** and **C)** and × 400 (**B** and **D**).

The expression and subcellular localization of the two H3K27 demethylases UTX and JMJD3 were further determined by immunohistochemical analysis in 63 paraffin-embedded RCC cancer tissues and paired adjacent tissues. The H3K27 demethylases were localized mainly in the nucleus in renal tissue. UTX and JMJD3 protein contents were higher in cancer tissues than in adjacent normal tissues (P < 0.01, Figures 3, 4, and 5).

## Discussion

Histone demethylases are known to be closely related to tumor formation [24] and several studies have also indicated an association between the H3K9 demethylase JMJD1A and RCC [25,26]. The role of histone demethylases in RCC is thus worthy of further investigation. Both UTX and JMJD3 are H3K27me3 demethylases and are essential for the expression of many genes, including *HOX*, through decreasing the levels of H3K27me3 [16,17]. Inactivating somatic mutations of *UTX* and reduced expression of *JMJD3* have been demonstrated in many cancers [21,27]. Both UTX and JMJD3 can be considered as candidate tumor suppressors [28,29], and may be involved in tumor suppression via an oncogene-induced senescence (OIS) mechanism.

The human tumor suppressor genes *INK4b-ARF-INK4a* are located on chromosome 9p21 and encode the proteins p16INK4a, p15INK4b and p14ARF. p16INK4a/p15INK4b are CDK4/CDK6 inhibitors and block retinoblastoma protein (Rb) phosphorylation and inactivation, which enhances the pRB-E2F signaling pathway. p14ARF inhibits the activity of p53-specific ubiquitin ligase murine double minute 2 (MDM2), which increases expression of p53 protein [30]. These three proteins are involved in senescence regulation and induction of cell cycle arrest at the G0/G1 phase, leading to cell senescence [31]. Cellular senescence causes irreversible growth arrest, while OIS is an important preventive mechanism for pre-cancerous damage [32] and can effectively block uncontrolled cell proliferation induced by DNA damage or oncogenic stimuli. Imbalance of OIS can lead to unlimited cell proliferation and eventually to cancer development [33].

The *INK4b-ARF-INK4a* locus is regulated by many factors, including histone modification [34]. The polycomb repressive complex 2 (PRC2) containing EZH2 can bind the *INK4b-ARF-INK4a* locus and silence their gene expressions through increasing local H3K27me3 content, which in turn promotes cell proliferation and reduces cell senescence [35,36]. In contrast, JMJD3 binding to the *INK4b-ARF-INK4a* locus can inhibit PRC2 occupancy and decrease H3K27me3 content, resulting in the increased expressions of these three proteins and promotion of cell senescence [29]. In primary Hodgkin’s lymphoma, JMJD3 is over-expressed and induced by the Epstein-Barr virus oncogene [37]. The results of our study revealed that H3K27me3 levels were lower in cancer tissues compared to adjacent normal tissues, accompanied by increased JMJD3 expression. Consistent with the strong decrease in H3K27me3 levels, *p16INK4a* gene expression was obviously higher in cancer tissues compared to normal tissues. Previous research indicated that homozygous deletions of the *INK4a/ARF* locus contributed to tumor progression in RCC [38]. These results suggest that inactivation or down-regulation of *p16INK4a* is a later event in RCC progression.

UTX also plays an important role in cell senescence in tumor suppression. The tumor suppressor Rb and its binding proteins are regulated by UTX-catalyzed H3K27me3 demethylation [39]. UTX can occupy the promoter region of *Rb* and the related gene *Rbl2* (retinoblastoma-like protein 2) and increase their expression, thus reducing cell proliferation and increasing cell senescence [40]. In Drosophila, *UTX*-mutant cells showed tumor-like growth characteristics accompanied by reduced *Rb* expression [41]. In the current study, UTX expression was obviously higher in cancerous tissues at both the mRNA and protein levels. Although all three of these proteins are upregulated in RCC, the reduction in H3K27me3 level implies that the H3K27 demethylases UTX and JMJD3 play more important roles than the H3K27 methyltransferase EZH2 in regulating *p16INK4a* expression.

Previous studies demonstrated that abnormal H3K27 levels or EZH2 expression were associated with cancer development and prognosis [14,42]. This study also demonstrated that upregulated expression of the H3K27 demethylases UTX and JMJD3 was relevant to tumor suppression. Previous studies found evidence for JMJD3 regulation in tissues from many cancers, including prostate cancer and primary Hodgkin’s lymphoma [20,37]. Further studies of the relationship between histone demethylases and cancer development will improve our understanding of the molecular mechanisms involved, and potentially aid in the development of new therapies for RCC [43,44]. The possible roles of UTX and JMJD3 in RCC can be summarized as follows: oncogene activation leads to increased binding of JMJD3 to the *p16INK4a* promoter and subsequent transcriptional induction through demethylation of H3K27me3 at the *INK4A-ARF* locus [31]. p16INK4a then inhibits RCC development via induction of cell cycle arrest. However, our understanding of the mechanism underlying cell senescence in tumor suppression is currently limited, and further studies are needed to clarify the roles of UTX and JMJD3 in RCC.

## Conclusions

In summary, this study revealed that upregulated expression levels of UTX and JMJD3 are common in cancer tissues in early stage RCC patients with a good prognosis. These H3K27 demethylases may inhibit cell proliferation in primary RCC through OIS. The results also imply that identification of the genes regulated by UTX and JMJD3 during RCC development will improve our understanding of the carcinogenesis and screening strategies in RCC. The potential roles of H3K27 demethylases as biomarker(s) for the early diagnosis of RCC and for prognostic evaluation need to be investigated.

## Competing interests

The authors declare that they have no competing interests.

## Authors’ contributions

XG, YC, AZ and XD were responsible for experimental design, data analysis and writing of manuscript. YS, XG and YW conducted the experiments including qRT-PCR, western blotting and immunohistochemical analysis. YS, WQ and AZ were responsible for collection and histological classification of clinical specimens. All authors have read and approved the final manuscript.

## Pre-publication history

The pre-publication history for this paper can be accessed here:

http://www.biomedcentral.com/1471-2407/12/470/prepub

## Supplementary Material

Additional file 1 **Figure S1.** The pathological stage of RCC. The A~D represents grade 1~4 of RCC according to the standard presented by Fuhrman et al [22]. click here for file
