# Supplementary figures and images for: Expression and significance of histone H3K27 demethylases in renal cell carcinoma
Source: BMC Cancer. 2012 Oct 12;12:470. doi: 10.1186/1471-2407-12-470 (PMC3520868; doi:10.1186/1471-2407-12-470)

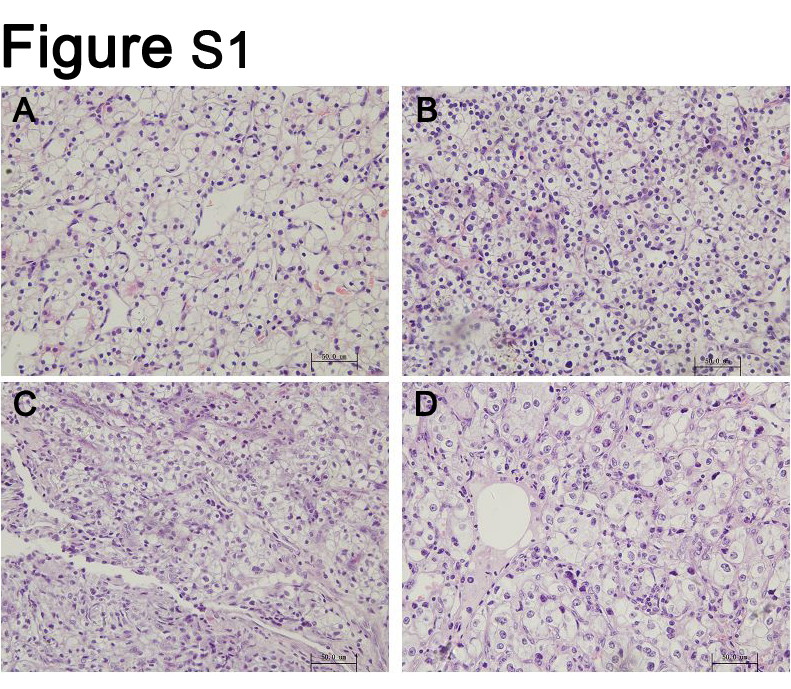

Supplement: Additional file 1 — Figure S1. The pathological stage of RCC. The A~D represents grade 1~4 of RCC according to the standard presented by Fuhrman et al [22]. [file 1471-2407-12-470-S1.tiff]
